# Root‐tip cutting and uniconazole treatment improve the colonization rate of *Tuber indicum* on *Pinus armandii* seedlings in the greenhouse

**DOI:** 10.1111/1751-7915.13511

**Published:** 2020-01-09

**Authors:** Xiaolin Li, Lei Ye, Xiaoping Zhang, Hao Tan, Qiang Li

**Affiliations:** ^1^ Soil and Fertilizer Institute Sichuan Academy of Agricultural Sciences Chengdu 610066 Sichuan China; ^2^ Key Laboratory of Coarse Cereal Processing Ministry of Agriculture and Rural Affairs College of Pharmacy and Biological Engineering Chengdu University Chengdu 610106 Sichuan China; ^3^ Present address: Key Laboratory of Coarse Cereal Processing Ministry of Agriculture and Rural Affairs 2025 # Chengluo Avenue Chengdu 610106 Sichuan China

## Abstract

The Chinese black truffle *Tuber indicum* is commercially valuable. The main factors influencing the success or failure of a truffle crop include the mycorrhizal colonization rate and host plant quality. The effects of a plant growth regulator (uniconazole) and plant growth management technique (root‐tip cutting) on *T. indicum* colonization rate and *Pinus armandii* seedling growth were assessed under greenhouse conditions. The results indicated that 10 mg l^−1^ uniconazole or the combination of 5 mg l^−1^ uniconazole and root‐tip cutting constitutes an effective method for ectomycorrhizal synthesis based on an overall evaluation of colonization rate, plant biomass, plant height, root weight, stem circumference and antioxidant enzyme activities (SOD and POD) of *P. armandii*. The abundance of Proteobacteria in the rhizosphere of colonized seedlings might serve as an indicator of stable mycorrhizal colonization. This research inspires the potential application of uniconazole and root‐tip cutting treatments for mycorrhizal synthesis and truffle cultivation.

## Graphical abstract

This study indicated that 10 mg l^−1^ uniconazole or the combination of 5 mg l^−1^ uniconazole and root tip cutting constitutes an effective method for ectomycorrhizal synthesis based on an overall evaluation of colonization rate, plant biomass, plant height, root weight, stem circumference, and antioxidant enzyme activities (SOD and POD) of *P. armandii*.

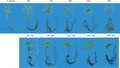

## Introduction

*Pinus armandii* Franch., an indigenous Chinese pine, is widely distributed in central and western China where it typically forms ectomycorrhizae with the Chinese black truffle *Tuber indicum* Cooke & Massee (Geng *et al.*, 2009; Li *et al.*, 2017). The ectomycorrhizal fungus *T. indicum* produces hypogeous fruiting bodies and is considered to be a culinary delicacy due to their unique flavours (Liu, 2007; Liu *et al.*, 2012; Cullere *et al.*, 2013; Vita *et al.*, 2015). As a delicious edible fungus, *T. indicum* is becoming more and more popular among consumers in Asia. Ectomycorrhizae of *P. armandii* with *T. indicum* were previously successfully synthetized in a greenhouse, preliminary steps to cultivating truffles (Geng *et al.*, 2009). Owing to decreases in wild truffle yield as a result of vegetation destruction, forest fires, improper harvesting and other human factors, the synthesis of truffle‐infected plants and truffle cultivation have attracted increasing attention in recent years. The efficient synthesis of ectomycorrhizae is considered to be the basis for the successful cultivation of truffles (Li *et al.*, 2017).

Root‐tip cutting is an effective technique used in plant growth management. It can alter the morphology, growth, root distribution, and architecture of the plant, and even increase the tolerance of the plant to abiotic stress (Mashela and Nthangeni, 2002; Chen *et al.*, 2014; Chen *et al.*, 2015). It was reported that a combination of root cutting and gibberellin A increases reproductive bud production in the conifer *Picea mariana* (Smith and Greenwood, 1995). Root redistribution via root cutting in agroforestry systems has confirmed that root cutting can serve as a potential tool for managing belowground competition when trees and crops are grown together (Wajja‐Musukwe *et al.*, 2008). However, how root‐tip cutting affects the colonization rate of the host plant by ectomycorrhizal fungi under greenhouse conditions remains unknown. Uniconazole is a well‐known plant growth regulator (Jiang *et al.*, 1998; Jiang *et al.*, 2008; He *et al.*, 2017) that regulates plant endogenous hormone levels and gene expression (Sasaki *et al.*, 2013; Liu *et al.*, 2015). The morphology and biomass allocation of *Salvia miltiorrhiza* was reportedly altered by uniconazole (Gao *et al.*, 2015). Uniconazole also plays an important role in improving crop production, increasing starch accumulation and enhancing primary root elongation and bolting delay (Huang *et al.*, 2015). Leaf‐spraying with uniconazole constitutes an important cultivation technique used in controlling flowering and improving the fruit setting of *Litchi chinensis*, as well as in enhancing the tolerance of soya bean to water deficit stress (Zhang *et al.*, 2007; Wei *et al.*, 2017). Proteomic and transcriptome analysis has revealed that key enzymes involved in endogenous hormone and chlorophyll biosynthesis, the regulation of flowering genes, and the expression levels of various transcription factors are regulated by uniconazole (Kojima *et al.*, 1996; Wijayanti *et al.*, 1996). The effect of uniconazole on mycorrhizal synthesis has not been previously reported.

Soil microorganisms, particularly rhizosphere microorganisms, play important roles in plant growth and development (Wang *et al.*, 2015; Esmaeili Taheri *et al.*, 2017; Mitter *et al.*, 2017). They can promote the growth of plants, increase the uptake of mineral elements and enhance the tolerance of plants to stress including drought stress, high salinity stress and disease stress (Abiala *et al.*, 2015; Bell *et al.*, 2015; Alegria Terrazas *et al.*, 2016; Baldrian, 2017). Bacteria in the soil or associated with truffle ascocarps were found to play important roles in truffle maturation and production of volatile organic compounds (Barbieri *et al.*, 2005; Antony‐Babu *et al.*, 2014; Splivallo *et al.*, 2015; Vahdatzadeh *et al.*, 2015). Mycorrhiza helper bacteria (MHB) are a group of organisms that could stimulate the formation of mycorrhizal symbiosis (Deveau and Labbé, 2006). Proteobacteria was found to be the largest and most diverse group in the ectomycorhizosphere of truffles (Li *et al.*, 2017). *Pseudomonas fluorescens* belonging to this group has been examined in previous studies to improve mycorrhization of truffle and plant growth (Dominguez *et al.*, 2012).

In this paper, the effects of different concentrations of uniconazole and root‐tip cutting treatments on the growth, morphology, distribution of dry biomass, antioxidant system and colonization rate of *P. armandii* seedlings by *T. indicum* were assessed. The effects of uniconazole and root‐tip cutting treatments on the bacterial community of the rhizosphere were also assessed. This paper provides useful insights on the potential application of uniconazole and root‐tip cutting treatments in ectomycorrhizal synthesis and the cultivation of commercial ectomycorrhizal fungi.

## Results

### Effects of *Tuber indicum* inoculation on seedling growth

**Figure 1:**
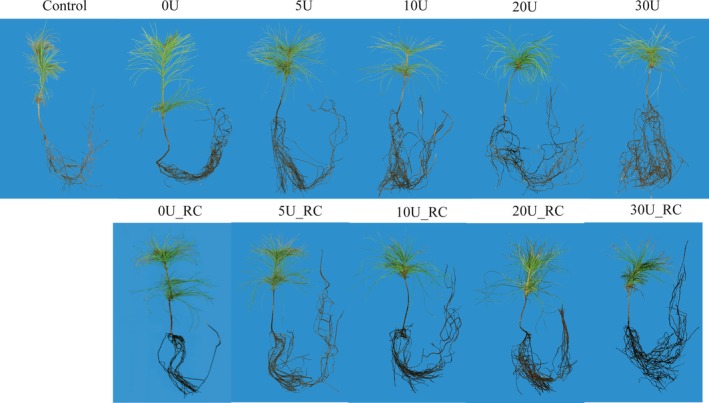
Morphological changes of *Pinus armandi* seedlings (7 months old) colonized by *Tuber indicum* under root‐tip cutting treatment and uniconazole treatment in the greenhouse. Control, *P. armandi* seedlings without *T. indicum* partner; U, *P. armandi* seedlings colonized by *T. indicum* sprayed with uniconazole (mg l^−1^); U_RC, *P. armandi* seedlings colonized by *T. indicum* treated with uniconazole (mg l^−1^) and root‐tip cutting.

**Figure 2:**
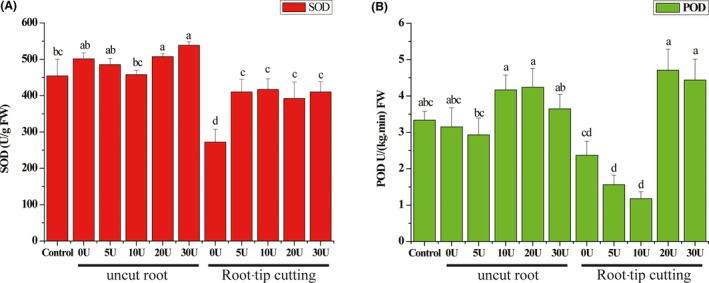
Effects of root‐tip cutting and different concentrations of uniconazole treatment (mg l^−1^) on superoxide dismutase (SOD) and peroxidase (POD) activities in roots of *Pinus armandi* seedlings (7 months old) colonized by *Tuber indicum* under greenhouse conditions. Control, *P. armandi* seedlings without *T. indicum* partner; U, *P. armandi* seedlings colonized by *T. indicum* sprayed with uniconazole (mg l^−1^). Each value is the mean of 10 replicates (±SD). Values followed by different lowercase letters indicate significant differences (*P* < 0.05) between samples.

**Figure 3:**
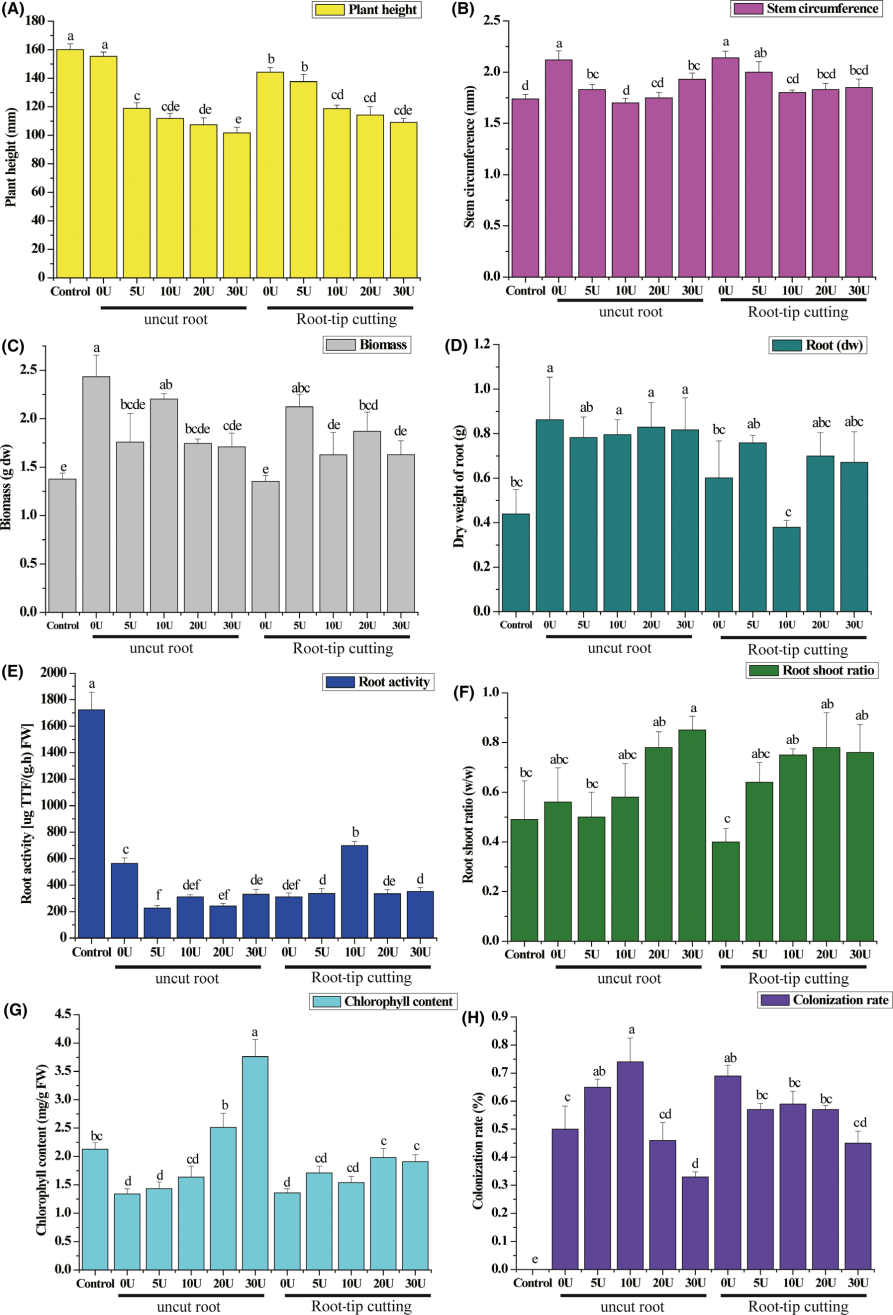
Effects of root‐tip cutting and different concentrations of uniconazole treatment (mg l^−1^) on plant height (A), stem circumference (B), biomass (C), root dry weight (D), root activity (E), root–shoot ratio (F), chlorophyll content (G) and colonization rate (h) of *Pinus armandi* seedlings colonized by *Tuber indicum* under greenhouse conditions. Control, *P. armandi* seedlings without *T. indicum* partner; U, *P. armandi* seedlings colonized by *T. indicum* sprayed with uniconazole (mg l^−1^). Each value is the mean of 10 replicates (±SD). Values followed by different lowercase letters indicate significant differences (*P* < 0.05) between samples.

Six months after spore inoculation, mycorrhization was successfully detected in *P. armandii* seedlings based on morphological and molecular evidence (KY296094 ITS sequence in GenBank; Fig. S1), while the 60 control seedlings were not colonized by truffles. The effects of *T. indicum* inoculation on the activities of root antioxidant enzymes (SOD and POD), plant height, stem circumference, biomass (dry weight, dw), root weight (dw), root–shoot ratio, dehydrogenase activity as index of the root activity and chlorophyll content of *P. armandii* seedlings were assessed in this paper (Fig. 1). SOD and POD activity, plant height and root–shoot ratio did not differ significantly between *P. armandii* seedlings inoculated with truffle and the control seedlings (Figs 2 and 3). Root activity and chlorophyll content decreased significantly in *P. armandii* seedlings after inoculation with truffle as compared with the control seedlings (*P* < 0.05). Inoculation of truffles on *P. armandii* seedlings exhibited a positive effect on the stem circumference, biomass and root weight, and was significantly higher compared to the control (*P* < 0.05).

### Effects of uniconazole on seedlings

The antioxidant enzyme activities of the roots of *P. armandii* colonized by *T. indicum* varied with the concentration of uniconazole (0, 5, 10, 20 and 30 mg l^−1^). Both SOD and POD activities decreased initially and then increased with the increase in uniconazole concentration (Fig. 2). The activity of SOD reached its lowest when the seedlings were sprayed with uniconazole at a concentration of 10 mg l^−1^ and then increased significantly when the concentrations of uniconazole reached 20 or 30 mg l^−1^ (*P* < 0.05). POD activity reached the minimum under the treatment of 5 mg l^−1^ uniconazole and increased significantly under 10 or 20 mg l^−1^ uniconazole treatment (*P* < 0.05).

The plant height, stem circumference and root activity of *P. armandii* seedlings colonized by *T. indicum* decreased significantly when uniconazole was applied (*P* < 0.05; Fig. 3). However, treatment with uniconazole displayed positive effects on the root–shoot ratio and chlorophyll content of the colonized seedlings, which increased with the concentration increase of uniconazole. Root biomass also increased under uniconazole in comparison to the untreated seedlings colonized by *T. indicum.* The biomass of *P. armandii* seedlings significantly decreased under uniconazole treatment, except at the concentration of 10 mg l^−1^. The colonization rate of *P. armandii* seedlings increased first and then decreased with increasing concentrations of uniconazole and peaked when the concentration was 10 mg l^−1^.

### Combined effects of root‐tip cutting and uniconazole treatment on seedlings

Superoxide dismutases (SOD) are a class of enzymes that catalyse the dismutation of superoxide into oxygen and hydrogen peroxide. As such, they constitute an important antioxidant defence in nearly all cells exposed to oxygen. The root‐tip cutting treatment significantly reduced SOD activities of colonized roots compared with the control roots and uncut colonized roots (*P* < 0.05; Fig. 2), with the exception of uncut colonized roots with 10 mg l^−1^ uniconazole application. The POD activity of the colonized roots from both uncut and cut seedlings decreased initially and then increased with an increase in uniconazole concentration, with the lowest activity recorded at a uniconazole concentration of 10 mg l^−1^.

The plant height, biomass, root weight and root activity of *P. armandii* seedlings with cut root tips colonized by *T. indicum* and without uniconazole application decreased significantly compared with uncut colonized seedlings (*P* < 0.05; Fig. 3). The stem circumference, root–shoot ratio and chlorophyll content of the colonized seedlings without uniconazole application were not significantly affected by root‐tip cutting. Root‐tip cutting had a positive effect on the colonization rate (*P* < 0.05).

A combined treatment of root‐tip cutting with 10 mg l^−1^ uniconazole application significantly reduced the biomass, root weight and colonization rate of *P. armandii* seedlings in comparison to those treated only with uniconazole (*P* < 0.05; Fig. 3). The root activity of *P. armandii* seedlings treated with root cutting and 10 mg l^−1^ uniconazole was significantly improved compared with those treated with only uniconazole, at all concentrations (*P* < 0.05). Root‐tip cutting treatment and uniconazole treatment at concentrations of 20 or 30 mg l^−1^ significantly reduced the chlorophyll content of the seedlings compared with those treated with uniconazole, at corresponding concentrations (*P* < 0.05).

### Combined effects of root‐tip cutting and uniconazole treatment on the bacterial diversity of the rhizosphere soil

Based on the Chao1 and ACE indices, the bacterial species richness in the rhizosphere soil of colonized seedlings decreased in comparison to those not inoculated with *T. indicum*, but this was not significant (Table 1). Conversely, a significant decrease (*P* < 0.05) in the Shannon diversity index was observed in the rhizosphere soil of the colonized seedlings compared to uninoculated seedlings. The Chao1 and species richness indexes were significantly reduced in the rhizosphere soil of colonized seedlings (*P* < 0.05) when the concentration of uniconazole reached 10 mg l^−1^ or higher in comparison with the rhizosphere of those not sprayed with uniconazole. The ACE index was significantly lowered (*P* < 0.05) in the rhizosphere soil of the seedlings under 30 mg l^−1^ uniconazole treatment compared to untreated plants. Root‐tip cutting and non‐cutting seemed to have no significant effect on the diversity of microorganisms in rhizosphere soil of colonized seedlings.

**Table 1:** Community richness and diversity indexes of bacteria in rhizosphere soil of *P. armandii* seedlings colonized by *Tuber indicum* under treatment with root‐tip cutting or different concentrations of uniconazole (mg l^−1^).

Treatment	Sample	Observed species	Chao1	ACE	Simpson	Shannon
Control	CK	1974 ± 72 a	1367 ± 67 a	1692.22 ± 161.75 a	0.99 ± 0.00 a	8.40 ± 0.03 a
Root‐tip cutting	0U	1872 ± 37 ab	1180 ± 75 ab	1649.13 ± 144.94 a	0.99 ± 0.00 ab	7.78 ± 0.15 b
	5U	1834 ± 31 ab	1055 ± 48 bc	1401.96 ± 124.03 ab	0.98 ± 0.01 c	7.40 ± 0.20 b
	10U	1525 ± 101 c	977 ± 91 c	1274.84 ± 193.18 bc	0.98 ± 0.01 abc	7.59 ± 0.17 b
	20U	1644 ± 169 bc	974 ± 48 c	1261.22 ± 102.00 bc	0.98 ± 0.01 bc	7.44 ± 0.15 b
	30U	1445 ± 23 c	880 ± 37 c	1012.79 ± 89.29 c	0.98 ± 0.02 ab	7.52 ± 0.09 b
Uncut root	0U	1789 ± 36 ab	1122 ± 41 ab	1628.72 ± 144.94 a	0.99 ± 0.00 ab	7.84 ± 0.11 b
	5U	1657 ± 82 bc	1061 ± 94 bc	1381.36 ± 124.03 ab	0.98 ± 0.00 c	7.56 ± 0.09 b
	10U	1532 ± 85 c	909 ± 58 c	1287.21 ± 193.18bc	0.98 ± 0.00 c	7.32 ± 0.12 b
	20U	1558 ± 127 c	893 ± 38 c	1190.06 ± 102.00 bc	0.98 ± 0.00 c	7.25 ± 0.23 b
	30U	1497 ± 87 c	824 ± 77 c	990.03 ± 89.29 c	0.98 ± 0.00 c	7.05 ± 0.22 b

CK, *P. armandii* seedlings without *T. indicum*; Root‐tip cutting, *P. armandii* seedlings colonized by *T. indicum* treated with root‐tip cutting; Uncut root, *P. armandii* seedlings colonized by *T. indicum* without root‐tip cutting; U, *P. armandii* seedlings colonized by *T. indicum* treated with uniconazole (mg l^−1^). Each value represents the mean of three replicates (±SD). Values followed by different lowercase letters indicate significant differences (*P* < 0.05) between samples in the same line.

### Taxonomic analyses of bacterial communities

**Figure 4:**
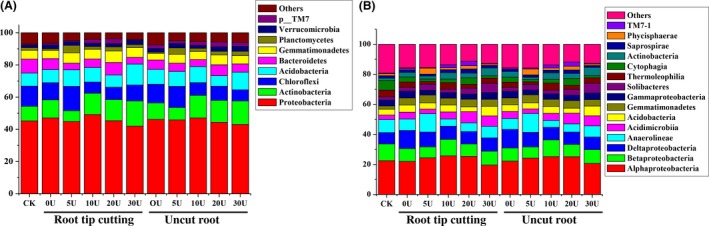
Effects of root‐tip cutting and different concentrations of uniconazole treatment (mg l^−1^) on bacterial communities in rhizosphere soil of *Pinus armandi* seedlings colonized by *Tuber indicum* in the greenhouse at the phylum (A) and class (B) levels. CK, *P. armandi* seedlings without *T. indicum* partner; root‐tip cutting, *P. armandii* seedlings colonized by *T. indicum* treated with root‐tip cutting; uncut root, *P. armandii* seedlings colonized by *T. indicum* without root‐tip cutting; U, *P. armandii* seedlings colonized by *T. indicum* treated with uniconazole (mg l^−1^). Each value is the mean of three replicates.

Each bacterial 16S rRNA gene sequence was taxonomically assigned from the level of phylum to genus based on the RDP 3 classifier (Fig. S2). A total of 34 phyla were identified in the rhizosphere soil of *P. armandii* seedlings with or without *T. indicum*, of which 21 were observed in all 18 samples (Fig. 4A). Four phyla, namely Proteobacteria, Actinobacteria, Chloroflexi and Acidobacteria were dominant across all samples with average abundances of 45.8%, 11.4%, 10.3% and 9.5% respectively. The abundance of these phyla exhibited no significant differences between the rhizosphere soil of cut colonized seedlings and that of uninoculated uncut seedlings. Proteobacteria constituted the most abundant phylum in the rhizosphere soil of cut colonized seedlings treated with 10 mg l^−1^ uniconazole, and its abundance decreased significantly when the uniconazole concentration was increased to 30 mg l^−1^ (*P* < 0.05).

Of the 59 classes detected, Alphaproteobacteria, Deltaproteobacteria, Betaproteobacteria and Anaerolineae were most abundant across all samples, with respective average values of 23.5%, 9.2%, 9.1% and 7.7% (Fig. 4B). The relative abundance of Deltaproteobacteria was significantly greater in the rhizosphere soil of colonized seedlings than the control seedlings (*P* < 0.05), while the cut colonized seedlings sprayed with 10 mg l^−1^ uniconazole contained the most Alphaproteobacteria.

**Figure 5:**
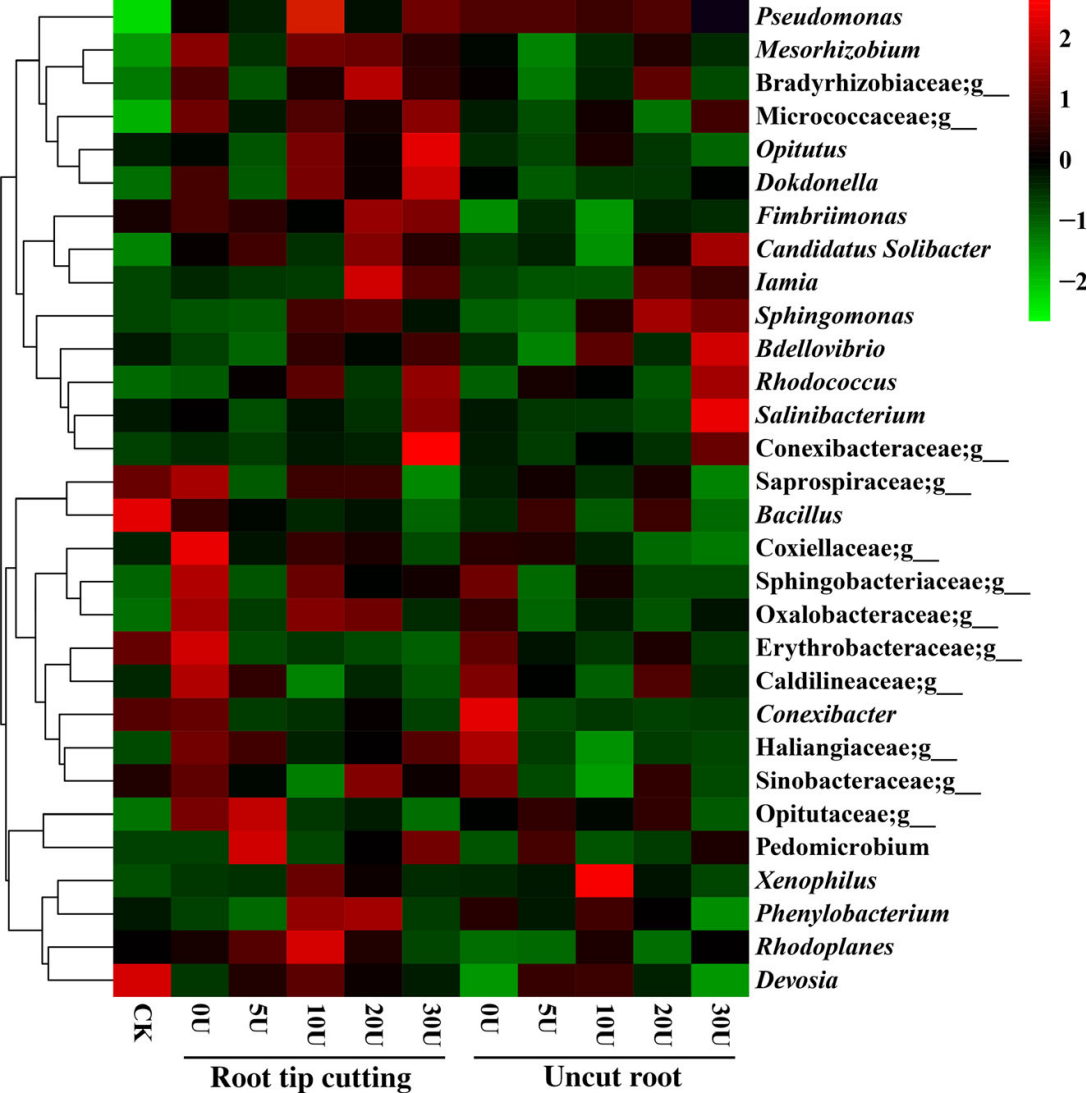
Heat‐map analysis of the 30 most abundant bacterial genera in rhizosphere soil of *Pinus armandi* seedlings colonized by *Tuber indicum* in the greenhouse. CK, *P. armandi* seedlings without *T. indicum* partner; root‐tip cutting, *P. armandii* seedlings colonized by *T. indicum* treated with root‐tip cutting; uncut root, *P. armandii* seedlings colonized by *T. indicum* without root‐tip cutting; U, *P. armandii* seedlings colonized by *T. indicum* treated with uniconazole (mg l^−1^). Each value is the mean of 3 replicates. The relative abundance of the sample at genus level increased with the colour block changing from green to red.

A total of 486 genera were detected, of which 145 were common to all samples (Fig. 5). *Rhodoplanes* (average 3.25%) and *Devosia* (2.63%) were most prominent in all samples, and *Devosia* was significantly more abundant in the rhizosphere soil of the control seedlings than colonized seedlings (*P* < 0.05). *Pseudomonas* was significantly more abundant in the rhizosphere soil of colonized seedlings than the control seedlings (*P* < 0.05). The cut colonized seedlings sprayed with 10 mg l^−1^ uniconazole contained the highest relative abundance of *Pseudomonas*.

**Figure 6:**
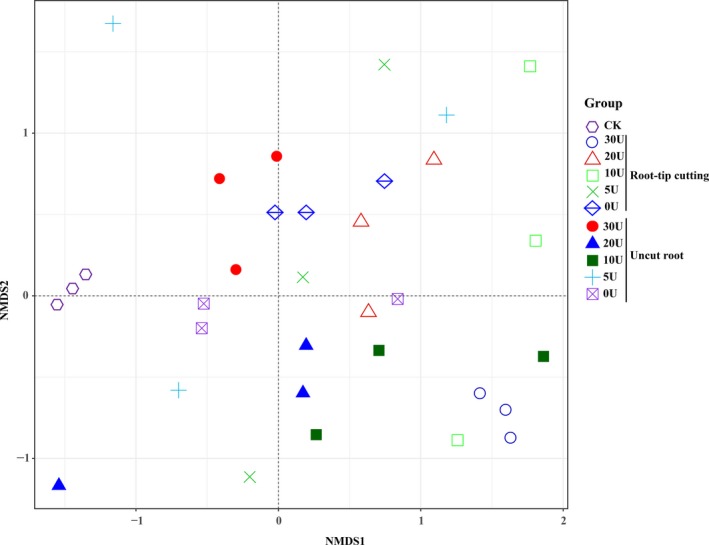
Nonmetric multidimensional scaling (NMDS) analysis of bacterial communities in rhizosphere soil of *P. armandii* seedlings colonized by *Tuber indicum* under different concentrations of uniconazole treatment (mg l^−1^). CK, *P. armandi* seedlings without *T. indicum* partner; root‐tip cutting, *P. armandii* seedlings colonized by *T. indicum* treated with root‐tip cutting; uncut root, *P. armandii* seedlings colonized by *T. indicum* without root‐tip cutting; U, *P. armandii* seedlings colonized by *T. indicum* treated with uniconazole (mg l^−1^). All experiments were conducted triple.

The differences in bacterial community compositions between the samples were visualized using nonmetric multidimensional scaling (NMDS) analysis. The bacterial community structure of the rhizosphere soil of the colonized seedlings sprayed with different concentrations of uniconazole differed significantly compared with the control treatments (Fig. 6), implying that the combination of root‐tip cutting and uniconazole treatment resulted in changes in the bacterial communities of the rhizosphere soil, which were initially from air and water.

## Discussion

The symbiosis of ectomycorrhizae has been well documented (Hogberg *et al.*, 2010; Torres‐Aquino *et al.*, 2017). Ectomycorrhizal fungi have been well studied, which could increase plant primary biomass and increase plant tolerance to stress (Abiala *et al.*, 2015; Bell *et al.*, 2015). (Boroujeni and Hemmatinezhad, 2015). In the present study, the effect of inoculation with *T. indicum* on the growth of seven‐month‐old *P. armandii* seedlings under greenhouse conditions was assessed. Inoculation of *T. melanosporum* has been proven to improve the growth and nutrient uptake of *Pinus halepensis* seedlings (Dominguez *et al.*, 2012). This is consistent with the results of this study whereby truffle inoculation was found to exhibit a positive effect on the stem circumference, biomass and root weight of *P. armandii* seedlings. Interestingly, inoculation with *T. indicum* reduced the root activity and chlorophyll content of the host seedlings under greenhouse conditions. An explanation for this negative effect on host physiology requires further analysis.

Uniconazole is a triazole chemical that is used as a plant growth retardant. It is active on a wide range of plants and acts by inhibiting the production of gibberellins (Liu *et al.*, 2015). In the present study, a colonization rate increase was observed in uncut colonized seedlings under 10 mg l^−1^ uniconazole treatment. Conversely, a concentration of 30 mg l^−1^ reduced the colonization rate. The uniconazole‐induced increase in root–shoot ratio, root length, increased root tips and increased root surface area (Fig. S1) under certain concentration was considered to be closely associated with the observed increase in colonization rate. The decrease in plant height and increase in chlorophyll content caused by uniconazole observed in this study is consistent with previous literature (Itoh *et al.*, 2005; He *et al.*, 2017). Biomass, root weight and antioxidant system (POD and SOD) activities of *P. armandii* inoculated with *T. indicum* were not significantly affected by 10 mg l^−1^ uniconazole treatment, which suggests potential application for the harvest of both plant (wood or fruit) and truffle (ascocarp). This increased colonization rate could be applied to the study of mycorrhizal synthesis and material exchange between mycorrhizal fungi and host plants. The effect of uniconazole on the artificial cultivation of truffle and fructification requires further verification in the field. Although 30 mg l^−1^ uniconazole treatment further increased the root–shoot ratio, the significant decrease in host plant height and biomass limited the increase in truffle colonization rate, as ectomycorrhizae rely on the host plant as a carbon source. According to previous research, the colonization rate and the quality of the host plant constitute the primary factors contributing to the success or failure of a truffle crop (Andres‐Alpuente *et al.*, 2014).

Root‐tip cutting is an efficient technique used in plant growth management. Its effects on the morphology, growth and colonization rate of the host plant were assessed in this paper. The results demonstrated that root‐tip cutting alone had a positive effect on colonization rate. The proliferation of secondary trophic roots caused by root‐tip cutting was also considered to be one of the main reasons for the increase of colonization rate. However, the significant decrease in plant height, biomass, root weight and root activity of root tip‐cut *P. armandii* seedlings colonized by *T. indicum* raises concerns about its further application in truffle cultivation. The activities of SOD and POD are considered to be important indicators for stress resistance in plants (Liu *et al.*, 2017; Tanveer and Shah, 2017), and in this study, these antioxidant enzymes were significantly reduced by root‐tip cutting. However, the combined treatment of 5 mg l^−1^ uniconazole and root‐tip cutting presents an alternative approach for mycorrhizal synthesis and was associated with relatively high colonization rates, biomass, plant height, root–shoot ratio, root weight and stem circumference of *P. armandii* seedlings.

Rhizosphere soil microorganisms play important roles in promoting plant growth and improving tolerance to disease and abiotic stress (Angela *et al.*, 2018). It was previously reported that the bacteria associated with truffle ascocarps contribute towards truffle volatile compositions (Splivallo *et al.*, 2015). Dominguez *et al. *(2012) reported that the additional inoculation of *P. fluorescens* CECT 844 doubled the rate of mycorrhization of *T. melanosporum* in comparison to a simple inoculation. The combined inoculation of *P. fluorescens* and *T. melanosporum* was found to improve the growth and nutrient uptake of the seedlings (Dominguez *et al.*, 2012). To further assess the effects of uniconazole and root‐tip cutting treatment on bacterial communities in the rhizosphere soil of the colonized seedlings, a high‐throughput sequencing method was used to analyse the rhizosphere soil bacterial community of the colonized seedlings under these treatments. Root‐tip cutting, uniconazole treatment and inoculation of *T. indicum* reduced the microbial diversity in the rhizosphere soil of *P. armandii* seedlings, particularly on some indicators, including the observed species, Chao1 and Shannon indexes. Our previous study found that inoculation of *T. indicum* decreased microbial diversity in the rhizosphere soil of *P. armandii* seedlings under the same conditions (Li *et al.*, 2017). Of all the major bacterial communities detected, three phyla, namely Proteobacteria, Actinobacteria and Chloroflexi, were dominant in all samples. Alphaproteobacteria and Deltaproteobacteria (both in the phylum Proteobacteria) were found to be more abundant in the rhizosphere soil of root tip‐cut or 10 mg l^−1^ uniconazole‐treated colonized seedlings than in control rhizosphere soil. Previous studies also found that α‐Proteobacteria and γ‐Proteobacteria comprised the predominant components of the bacterial communities of truffles (Barbieri *et al.*, 2007; Vahdatzadeh *et al.*, 2015; Li *et al.*, 2017). Previous studies have demonstrated that *Pseudomonas fluorescens* (Proteobacteria) improved the establishment and functioning of ectomycorrhizal symbiosis (Dominguez *et al.*, 2012). The close association of these communities with the presence of truffle mycelia is thought to be important for the growth and mycorrhizal synthesis of truffles (Li *et al.*, 2017). The high abundance of Proteobacteria in the rhizosphere soil of root tip‐cut or 10 mg l^−1^ uniconazole‐treated colonized seedlings may be used as an indicator of colonization of ectomycorrhizal fungi and inspires potential application in ectomycorrhizal fungi cultivation.

In summary, *T. indicum* inoculation exhibits a positive effect on the stem circumference, biomass and root weight of *P. armandii* seedlings. 10 mg l^−1^ uniconazole or the combination of 5 mg l^−1^ uniconazole and root‐tip cutting constitutes an improved method for truffle ectomycorrhizal synthesis, which was associated with relatively high colonization rates, biomass, plant height, root–shoot ratio, root weight and stem circumference of *P. armandii* seedlings. Root‐tip cutting, uniconazole treatment and inoculation of *T. indicum* reduced the microbial diversity in the rhizosphere soil of *P. armandii* seedlings, particularly on some indicators, including the observed species, Chao1 and Shannon indexes. However, the high abundance of Proteobacteria presented in the rhizosphere soil of root tip‐cut or 10 mg l^−1^ uniconazole‐treated colonized seedlings may be used as an indicator of colonization of truffle. This research inspires the potential application of uniconazole and root‐tip cutting treatments for ectomycorrhizal synthesis and truffle cultivation.

## Experimental procedures

### *Pinus armandii* seedling cultivation and root‐tip cutting treatment

*Pinus armandii* seedlings were cultivated in a greenhouse according to our previously described method (Li *et al.*, 2017). Briefly, *P. armandii* seeds, purchased from a local seed company (Tianhe seed company, Lianyungang, Jiangsu), were surface sterilized with 30% H_2_O_2_ for 30 min and washed three times with sterile water. Surface‐sterilized seeds were sown in plastic containers filled with sterilized substrate (vermiculite, perlite and water at a ratio of 1:1:1, v/v/v), which were autoclave‐sterilized for 90 min at 121°C to prevent contamination. One month later, *P. armandii* seedlings of approximately the same height and exhibiting healthy growth were selected to test the effects of root‐tip cutting on plant growth and mycorrhizal synthesis. They were removed from the plastic container, taking care to maintain root integrity, and the distal part of taproots (1 cm in length) was excised using sterilized scissors. The root tips of the control treatment were left unaltered. Both the treatment and the control seedlings were transplanted into containers with 1 L sterilized substrate (peat, vermiculite, organic soil and water at a ratio of 1:1:1:0.9, v/v/v/v) for *T. indicum* inoculation and uniconazole treatment. The organic soil used in this experiment was purchased from Klasmann‐Deilmann China Ltd. (Shanghai, China). The final pH of the homogenized substrate was adjusted to 7.5 by adding calcium hydroxide (Geng *et al.*, 2009).

### Truffle inoculation and uniconazole treatment

Truffle inoculation was performed according to our previously described method (Li *et al.*, 2017). Briefly, the spore inoculum of *T. indicum* was obtained by blending the ascocarps, which had been surface sterilized with 75% alcohol, and soaking in sterile water. The ascocarps were collected from the field when they were completely mature and were then identified by morphological and ITS‐rDNA sequence analysis (Wang *et al.*, 2006). Five millilitre sterile water mixed with 2 g spore powder was inoculated to the root surface of seedlings through sterile syringes (about 2 × 10^7^ spores per plant). Sixty *P. armandii* seedlings without pruned roots and watered with 5 ml sterile water served as controls. The remaining 600 seedlings were inoculated with truffle spores (including 300 with pruned root tips and 300 without pruned roots) and sprayed with different concentrations of uniconazole (0, 5, 10, 20 and 30 mg l^−1^). Wettable powder (5%), which has been shown to have no effect on plant growth (Kabir *et al.*, 2016), was used to improve the uniformity of the uniconazole in distilled water. Total volumes of 10 ml of different concentrations of uniconazole were sprayed on the needles of each seedling, and each treatment consisted of sixty seedlings. All pots were maintained in the greenhouse under uniform conditions (the daytime temperature of the greenhouse was 20–25°C; the night temperature was 18–20℃C; natural light; air humidity was 60–80%), with 5 m distance between different treatment groups. The seedlings were initially watered 7 days following uniconazole spraying, after which they were watered every 3 days. No fertilizers were added to the plants. After 6 months, the mycorrhizae of the *P. armandii* seedlings colonized by *T. indicum* were detected by morphological and molecular analysis using a microscope and ITS‐rDNA sequence analysis (Geng *et al.*, 2009; Li *et al.*, 2017). The seedlings, roots and rhizosphere soil were harvested for antioxidase activity assay (SOD and POD), plant morphometry (plant height, stem circumference and root–shoot ratio), root activity (dehydrogenase activity), chlorophyll content, colonization rate detection and soil microbial community determination using high‐throughput sequencing.

### Determination of antioxidant enzyme activity in the roots

Harvested *P. armandii* roots (0.1 g for each seedling) were first ground in liquid nitrogen with a pestle and the resultant paste soaked in 100 mM phosphate buffer (pH 7.5) containing 1 mM EDTA and 1% polyvinylpyrrolidone (w/v) at 4°C. The homogenate was centrifuged at 8000 *g* at 4°C for 10 min, and then, the supernatant was centrifuged at 12 000 *g* at 4°C for 20 min. This final supernatant was used to measure the enzyme activity.

SOD activity of root extracts was determined by the inhibition of the photochemical reduction of nitroblue tetrazolium (NBT) following the method of Fridovich (2011). Peroxidase (POD) activity was measured using a peroxidase assay kit (Nanjing Jiancheng Bioengineering Institute, Nanjing, Jiangsu, China) according to the manufacturer’s instructions. Using a spectrophotometer (MODEL U‐3900/3900H; Hitachi High‐Technologies Corporation, Tokyo, Japan), one unit of enzyme activity (1 U) was defined as the change in 0.01 absorbance units per minute at 460 nm.

### Determination of plant growth and colonization rate

The plant height and stem circumference of *P. armandii* seedlings were measured using ruler and vernier caliper respectively. The biomass of each plant was assessed based on the aboveground and belowground dry matter weight. For dry weight determination, the seedlings were first washed to remove any substrate attached to the roots, after which they were oven‐dried at 105°C for 30 min to halt respiration and then oven‐dried at 75°C to achieve constant weight (Maunoury‐Danger *et al.*, 2010). The dry weight of the shoots and roots was measured once the material had cooled. The root–shoot ratio was expressed as the ratio of dry weight between the belowground and aboveground parts of the plant.

Root activity was measured using the triphenyl tetrazolium chloride (TTC) method (Zhang *et al.*, 2012). When TTC is added to a tissue, it can be reduced by dehydrogenase, primarily succinate dehydrogenase. Briefly, 0.1 g fresh root material for each seedling was immersed in 10 ml of a mixed solution comprising equal volumes of 0.4% TTC solution and phosphate buffer. The mixture was kept in the dark at 37°C for 2 h, after which 2 ml of 1 M H_2_SO_4_ was added to stop the reaction in the roots. The roots were blotted with filter paper and then transferred into a mortar and ground to a fine powder in 3–4 ml ethyl acetate. The resulting red powder was transferred into a volumetric flask, and ethyl acetate was added to adjust the total volume to 10 ml. The absorbance of the extract at 485 nm was measured. Root activity was expressed as the TTC reduction intensity. Root activity = amount of TTC reduction (µg)/fresh root weight (g) × time (h) (Zhang *et al.*, 2012).

The chlorophyll content was measured using a handheld chlorophyll meter (SPAD‐502; Konica Minolta Company, Tokyo, Japan, measuring area: 2 × 3 mm). The same sections of the needles taken from the centre of the seedlings were selected for analysis. About 15 needles were analysed for chlorophyll content for each seedling.

Root colonization by *T. indicum* under different treatments was quantified. The root segments colonized by *T. indicum* were counted under a stereoscope based on the mycorrhizal fungal structures. A total of 30 root segments (each segment was about 1 cm long) were randomly selected and observed for each seedling (Andres‐Alpuente *et al.*, 2014). Mycorrhizal colonization rate was expressed as: mycorrhizal colonization rate (%) = (root segments colonized by *T. indicum*/total root segments observed) × 100 (Benucci *et al.*, 2012).

### Soil microbial DNA extraction and HiSeq sequencing

Soils at the interface with the ectomycorrhizae or non‐mycorrhizal roots (soil adhering to the root tip) were collected using needles and forceps. About 0.2 g of rhizosphere soil was taken from each seedling, and all experiments were conducted in triplicate. Total DNA of the microorganisms in the rhizosphere soil of *P. armandii* was extracted using a soil DNA kit (D5625‐01; Omega Bio‐tek Inc., Norcross, GA, USA) according to the manufacturer's instructions. The concentration and integrity of the extracted DNA were monitored on 1% agarose gels and determined using a Qubit ® 2.0 fluorometer (Invitrogen, Shanghai, China). The DNA was diluted to 1 ng μl^−1^ using sterile water.

The 16S V4 genes of all the samples were amplified using the universal primers 515F‐806R with the barcode as a marker for distinguishing samples (Fu *et al.*, 2016; Li *et al.*, 2017). PCRs were conducted according to Li *et al* *. * (2017) using Phusion® High‐Fidelity PCR Master Mix (New England Biolabs, Ipswich, MA, UK). PCR products obtained from three technical replicates were combined in equimolar ratios for each sample and purified with a Qiagen Gel Extraction Kit (Qiagen, Holden, Germany). PCR amplicon libraries were generated using a TruSeq® DNA PCR‐Free Sample Preparation Kit (lllumina, San Diego, CA, USA) in accordance with the instructions, and index codes were added. The library quality was assessed on a Qubit® 2.0 fluorometer (Thermo Scientific, Shanghai, China) and Agilent Bioanalyzer 2100 system. Sequencing was carried out on an Illumina HiSeq 2500 platform. All the experiments contained three biological repeats.

### Sequencing data analysis

The sequence reads were assigned to samples based on their respective barcodes and truncated by cutting off the barcode and primer sequence (BioProject: PRJNA391233). Reads that overlapped, which were generated from the opposite end of the same DNA fragment, were merged using FLASH (Magoc and Salzberg, 2011). Quality filtering of the raw tags (Caporaso *et al.*, 2010; Bokulich *et al.*, 2013) was performed under filtering conditions that were selected to obtain high‐quality clean tags according to the QIIME quality control process (Table S1). The tags were compared with the reference database using the UCHIME algorithm to detect and remove chimeric sequences (Edgar *et al.*, 2011). Sequences were assigned to the same operational taxonomic units (OTUs) with ≥ 97% similarity using UPARSE software (Edgar, 2013). Taxonomic information of the OTUs was annotated using the Greengenes Database based on Ribosomal Database Project (RDP) 3 classifier (Cole *et al.*, 2009). Rarefaction curves were used to estimate coverage. Alpha Diversity (species richness, Chao1), ACE (http://www.mothur.org/wiki/Ace), Simpson and Shannon indexes, and beta diversity were calculated with qiime (version 1.7.0). The differences in bacterial community compositions between the samples were visualized by nonmetric multidimensional scaling (NMDS) analysis (Schneider and Bissett, 1981) using r software.

### Statistical analysis

The data of this study are presented as means ± standard deviation (SD) of at least three biological replicates for each treatment. Statistical analysis was carried out by multivariate analysis of variance using spss 19.0 (Armonk, NY, USA). Least significant difference (LSD) was performed to test if the results of multivariate analysis of variance between the different treated groups were significant at *P* < 0.05.

## Conflict of interest

None declared.

## Supporting information


**Fig. S1**. Roots of *Pinus armandii* seedlings colonized by *Tuber indicum *under root‐tip cutting and different concentrations of uniconazole treatment (mg l^−1^). Control, *P. armandi* seedlings without *T. indicum* partner; U, *P. armandi *seedlings colonized by* T. indicum* sprayed with uniconazole (mg l^−1^); U_RC, *P. armandi *seedlings colonized by *T. indicum* treated with uniconazole (mg l^−1^) and root‐tip cutting.
**Fig. S2**. Rarefaction curves for bacterial operational taxonomic units (OTUs) in different samples (cut‐off value at 97% similarity). In the rarefaction curves, the number of OTUs increased with sequencing reads. Control, *P. armandi* seedlings without *T. indicum* partner; RC, *P. armandi* seedlings colonized by *T. indicum *treated by root‐tip cutting; U_RC, *P. armandi *seedlings colonized by *T. indicum* treated with uniconazole (mg l^−1^) and root‐tip cutting. All experiments were conducted triple.
**Table S1**. Throughput and quality of Hiseq sequencing of bacterial communities in rhizosphere soil of *Pinus armandi* seedlings colonized by *Tuber indicum* in the greenhouse.
