# Supplementary material for: Root‐tip cutting and uniconazole treatment improve the colonization rate of Tuber indicum on Pinus armandii seedlings in the greenhouse
Source: Microb Biotechnol. 2020 Jan 9;13(2):535–47. doi: 10.1111/1751-7915.13511 (PMC7017816; doi:10.1111/1751-7915.13511)
Supplement: Supplementary file 1 — Fig. S1. Roots of Pinus armandii seedlings colonized by Tuber indicum under root‐tip cutting and different concentrations of uniconazole treatment (mg l−1). Control, P. armandi seedlings without T. indicum partner; U, P. armandi seedlings colonized by T. indicum sprayed with uniconazole (mg l−1); U_RC, P. armandi seedlings colonized by T. indicum treated with uniconazole (mg l−1) and root‐tip cutting. Fig. S2. Rarefaction curves for bacterial operational taxonomic units (OTUs) in different samples (cut‐off value at 97% similarity). In the rarefaction curves, the number of OTUs increased with sequencing reads. Control, P. armandi seedlings without T. indicum partner; RC, P. armandi seedlings colonized by T. indicum treated by root‐tip cutting; U_RC, P. armandi seedlings colonized by T. indicum treated with uniconazole (mg l−1) and root‐tip cutting. All experiments were conducted triple. Table S1. Throughput and quality of Hiseq sequencing of bacterial communities in rhizosphere soil of Pinus armandi seedlings colonized by Tuber indicum in the greenhouse. [file MBT2-13-535-s001.docx]

**Figure S1** Roots of *Pinus armandii* seedlings colonized by *Tuber indicum* under root-tip cutting and different concentrations of uniconazole treatment (mg/L). Control, *P. armandi* seedlings without *T. indicum* partner; U, *P. armandi* seedlings colonized by *T. indicum* sprayed with uniconazole (mg/L); U_RC, *P. armandi* seedlings colonized by *T. indicum* treated with uniconazole (mg/L) and root-tip cutting.

**Figure S2** Rarefaction curves for bacterial operational taxonomic units (OTUs) in different samples (cut-off value at 97% similarity). In the rarefaction curves, the number of OTUs increased with sequencing reads. Control, *P. armandi* seedlings without *T. indicum* partner; RC, *P. armandi* seedlings colonized by *T. indicum* treated by root-tip cutting; U_RC, *P. armandi* seedlings colonized by *T. indicum* treated with uniconazole (mg/L) and root-tip cutting. All experiments were conducted triple.

**Table S1** Throughput and quality of Hiseq sequencing of bacterial communities in rhizosphere soil of *Pinus armandi* seedlings colonized by *Tuber indicum* in the greenhouse.

| Sample | Raw Reads | Clean Reads | Proportion |
| --- | --- | --- | --- |
| Control1 | 51536 | 33412 | 64.83% |
| Control2 | 61748 | 40044 | 64.85% |
| Control3 | 43197 | 27282 | 63.16% |
| 0U_RC1 | 54984 | 37471 | 68.15% |
| 0U_RC2 | 57163 | 39561 | 69.21% |
| 0U_RC3 | 81006 | 52209 | 64.45% |
| 5U_RC1 | 52994 | 38825 | 73.26% |
| 5U_RC2 | 62734 | 46627 | 74.32% |
| 5U_RC3 | 75088 | 50557 | 67.33% |
| 10U_RC1 | 33773 | 25610 | 75.83% |
| 10U_RC2 | 57122 | 36440 | 63.79% |
| 10U_RC3 | 32592 | 24288 | 74.52% |
| 20U_RC1 | 68486 | 46321 | 67.64% |
| 20U_RC2 | 65730 | 45652 | 69.45% |
| 20U_RC3 | 41038 | 32325 | 78.77% |
| 30U_RC1 | 35675 | 26008 | 72.90% |
| 30U_RC2 | 42110 | 22152 | 52.61% |
| 30U_RC3 | 39201 | 26993 | 68.86% |

Control, *P. armandi* seedlings without *T. indicum* partner; U_RC, *P. armandi* seedlings colonized by *T. indicum* treated with uniconazole (mg/L) and root-tip cutting. All experiments were conducted triple.
